# Unraveling the Impact of *Dab1* Gene Silencing on the Expression of Autophagy Markers in Lung Development

**DOI:** 10.3390/life14030316

**Published:** 2024-02-28

**Authors:** Azer Rizikalo, Mirko Maglica, Nela Kelam, Ilija Perutina, Marin Ogorevc, Anita Racetin, Natalija Filipović, Yu Katsuyama, Zdenka Zovko, Josip Mišković, Katarina Vukojević

**Affiliations:** 1 Department of Anatomy, School of Medicine, University of Mostar, 88000 Mostar, Bosnia and Herzegovina; azer.rizikalo@mef.sum.ba (A.R.); mirko.maglica@mef.sum.ba (M.M.); ilija.perutina@mef.sum.ba (I.P.); natalija.filipovic@mefst.hr (N.F.); zdenka.zovko@mef.sum.ba (Z.Z.); josip.miskovic@mef.sum.ba (J.M.); 2 Department of Anatomy, Histology and Embryology, University of Split School of Medicine, 21000 Split, Croatia; nela.kelam@mefst.hr (N.K.); marin.ogorevc@mefst.hr (M.O.); amuic@mefst.hr (A.R.); 3 Department of Medical Genetics, School of Medicine, University of Mostar, 88000 Mostar, Bosnia and Herzegovina; 4 Department of Anatomy, Shiga University of Medical Science, Otsu 520-2192, Japan; kats@belle.shiga-med.ac.jp; 5 Center for Translational Research in Biomedicine, University of Split School of Medicine, 21000 Split, Croatia

**Keywords:** Lc3b, Grp78, Hsc70, mTOR, Lamp2a, *yotari*, autophagy, lung injury

## Abstract

The purpose of this study was to evaluate the effects of *Dab1* gene silencing on the immunoexpression of light chain 3 beta (Lc3b), glucose regulating protein 78 (Grp78), heat shock cognate 71 (Hsc70), mammalian target of rapamycin (mTOR) and lysosomal-associated membrane protein 2A (Lamp2a) in the lung tissue of developing *yotari* (*Dab1*^−/−^) and wild-type (wt) mice. The lung epithelium and mesenchyme of the embryos at gestational days E13.5 and E15.5 were examined using immunofluorescence and semi-quantitative methods. In the pulmonary mesenchyme and epithelium, Grp78 and Lc3b of moderate fluorescence reactivity was demonstrated in wt mice for both evaluated time points, while *yotari* mice exhibited only epithelial reactivity for the same markers. Mild punctate expression of Hsc70 was observed for both genotypes. A significant difference was present when analyzing mTOR expression, where wt mice showed strong perinuclear staining in the epithelium. According to our data, *Dab1* gene silencing may result in autophagy abnormalities, which could then cause respiratory system pathologies via defective lung cell degradation by lysosome-dependent cell elimination.

## 1. Introduction

*Yotari* mutant mice, generated by spontaneously acquiring a mutation in the *Dab1* gene, have histological abnormalities of the central nervous system that are remarkably similar to those of reeler (*reelin*^−/−^) mice. This suggests that *Dab1* and *Reelin* are part of the same signaling pathway [1,2]. Reelin expression has been reported in mouse and human fetal tissue [3]. It has also been shown that depleting reelin reduces the leukocyte recruitment in the circulation of several organs [4,5]. Autophagy is essential for the pathogenesis and development of many chronic lung diseases as well as for the proper functioning of the lung’s inflammatory response system [6]. The multistage process of lung development in mouse embryos consists of three essential phases. Pseudoglandular stage: cuboidal epithelial cells lined with epithelial tubes undergo branching morphogenesis to resemble exocrine glands. This stage begins on the tenth developmental day. The respiratory tree continues to grow in length and diameter throughout the canalicular stage, which begins on the sixteenth developmental day and is characterized by vascularization and angiogenesis along the airway, as well as the terminal saccular stage, which consists of a significant interstitium thinning and ends at approximately the fifth postnatal day [7,8].

When lung cells are exposed to stressors such as oxidants, hypoxia, inflammation, ischemia-reperfusion, endoplasmic reticulum (ER) stress, pharmaceuticals, or inhaled xenobiotics (smoke from cigarettes or air pollution), autophagy may be a general inducible adaptive response that helps the cells recover [9]. It also has an important role in pathological conditions such as α1-antitrypsin deficiency, pulmonary hypertension, cystic fibrosis, acute lung injury, and interstitial lung disease [10].

Christian de Duve originally mentioned autophagy, which translates as “eating of self”, more than 50 years ago. His main evidence for this theory came from the observations of mitochondrial degradation within lysosomes in rat models [11]. Genetic screening has currently identified 32 different autophagy-related genes (Atg) in yeast. Interestingly, many of these genes are conserved in slime mold, plants, worms, flies, and mammals, highlighting the significance of the autophagic process in responses to starvation throughout phylogeny [12]. Autophagy can be broadly classified into three categories: chaperone-mediated autophagy (CMA), microautophagy, and macroautophagy. The beginning phase, or phagophore creation, is the first stage of macroautophagy, which is the most well-studied kind of autophagy. The phagophore membrane stretches in a subsequent elongation phase to encircle, target, and engulf the cytoplasmic cargo to form an autophagosome, that fuses with the lysosome to form an autolysosome. In contrast, cytosolic materials are immediately absorbed by the lysosome during microautophagy due to lysosomal membrane invagination. Large structures can be engulfed by both macro- and microautophagy via both non-selective and selective mechanisms. However, selectivity has been associated with chaperone-mediated autophagy (CMA), which is a dynamic process that requires the selective transport of proteins to the lysosomes. Not all proteins can undergo degradation via CMA. For them to be CMA substrates, containing a specific amino acid sequence (KFERQ-like motif) is mandatory. This motif binds to a cytosolic chaperone (such as Hsc70), making a complex that is recognized by the lysosomal membrane receptor called lysosomal-associated membrane protein 2a (Lamp2a), resulting in internalization and rapid intralysosomal degradation of selected proteins [13,14,15]. Numerous essential genes in human genome linked to autophagy have already been discovered, along with the products they produce, which control specific phases in the activation or progression of the autophagic pathway [16].

According to our most recent studies, the renal immunoexpression pattern in *yotari* mice varies significantly from wild-type mice in terms of the expression of numerous markers (autophagic, primary cilia, etc.), which may indicate that these mechanisms play an essential role in nephrogenesis [17,18].

One of the most well-known autophagy-related proteins is microtubule-associated protein light chain 3 (Lc3), which is often utilized as a specific marker for monitoring macroautophagy activity [19]. It requires the activity of two ubiquitin-like conjugation systems for autophagosome formation to occur. The conversion of Lc3 from its free form, Lc3-I, to its phosphatidylethanolamine-conjugated form, Lc3-II, is considered a crucial stage in the creation of autophagosomes [20,21]. Lc3-II levels therefore correlate well with autophagosome number, making it a practical and reliable marker to assess changes in autophagic activity. It has thus far been utilized to probe for autophagic flux, which is a measure of autophagic degradation activity [22].

Lysosomal-associated membrane protein 2a (Lamp2a) is localized in type II alveolar cells (pneumocytes) of the lungs in mice. In type II pneumocytes, the Lamp protein family is localized in lamellar bodies, secretory organelles releasing pulmonary surfactant into the extracellular space to lower surface tension at the air/liquid interface [23]. In CMA, cellular components are delivered and degraded by a chaperone-dependent selective process [24]. Lamp2a functions as a channel and receptor for the distribution of cytosolic proteins. Lamp-1 and Lamp-2 are thought to make up around half of all the proteins in the lysosomal membrane. It is surprising to learn that mice lacking either Lamp-1 or Lamp-2 can survive. On the other hand, mice lacking in both Lamp-1 and Lamp-2 have an embryonic lethal phenotype. Lamp-2 seems to have more specialized roles since the consequences of a Lamp-2 single defect are greater than those of a Lamp-1 single deficiency [25,26]. Also, mice lacking in autophagy suffer spontaneous sterile lung inflammation, which is defined by a substantial infiltration of inflammatory cells, the thickening of the submucosa, metaplasia of goblet cells, and elevated collagen levels [27].

Rapamycin, a serine/threonine kinase that has managed to be conserved throughout evolution, regulates various cellular functions, including apoptosis, autophagy, translation, metabolism, and inflammation. It also serves as a sensor of cellular nutrition and energy status in the environment. Numerous mitogens, growth factors, and nutrients trigger the activation of mammalian target of rapamycin complexes (mTORC1 and mTORC2) which govern processes like autophagy, innate and adaptive immune response, cell growth, proliferation, and development. Autophagy is suppressed by mTORC1; therefore, it is dramatically induced by mTORC1 inhibition [28]. Interestingly, mTOR signaling has also been linked to cigarette-induced chronic obstructive pulmonary disease (COPD) and emphysema. In wild-type (wt) mice exposed to smoke, the mTOR inhibitor rapamycin showed a protective role by lowering alveolar inflammation [29].

The integrity of the ER is maintained by glucose-regulated protein 78 (Grp78), a member of the heat shock protein 70 (Hsp 70) family. In general, Grp78 is thought to be a significant ER chaperone that controls transmembrane ER inducers and promotes protein folding. It is mostly found in the lumen of the ER and is enhanced during the initial phases of stress [30,31]. It can be induced in fibroblasts and normal or transformed lung cells as a general reaction to smoke from cigarettes or substances linked to lung damage, like hyperoxia and bacterial inflammation in mice [32]. A recent study has indicated that pulmonary conditions such as acute lung damage, idiopathic pulmonary fibrosis, and neutrophilic asthma are associated with increased levels of Grp78 [33].

The amino acid sequence of heat shock cognate 71 kDa protein (Hsc70) varies from that of Hsp70 by just 25% [34]. The ability of Hsc70 to transport proteins between the cytoplasm and organelles is one of its functions in preserving cellular homeostasis [35]. It also performs a unique role of promoting the import/export of various client proteins into the nucleus while switching between the cytoplasm and nucleus space [36]. Heat shock proteins (Hsps) are essential chaperones in cells that perform a variety of maintenance and repair tasks, such as controlling proteostasis. They have also been linked to the development of pulmonary fibrosis in animal models [37].

In mice, the budding of an epithelial tube marks the beginning of lung development around the ninth day of embryonic life. A decrease in cell proliferation and the appearance of highly differentiated cell types, such as type I and type II pneumocytes in the distal lung, are signs of late lung maturation during the sixteenth embryonic day [38]. Previous research discovered that “alveolar developmental disorders” would occur in mice whose expression of certain corresponding genes was significantly higher during their growth and development. Depletion of Dab1/reelin has been shown to reduce the recruitment of circulating leukocytes into several organs [4,39]. The cytokine profiles of mice with Dab1/reelin depletion differ significantly from their homozygous or heterozygous wt littermates [40]. Cells can undergo structural remodeling during differentiation and development, resist environmental deprivation, and even delay aging by eliminating defective organelles as part of autophagy’s housekeeping functions. We predicted that the *Dab1* mutant animals in this work would exhibit a different pattern of immunoexpression of autophagy markers to those of wt animals since this mechanism is crucial for proper lung growth [41]. In regard to the causes of lung pathologies in *yotari* mice, this work sought to investigate the effects of *Dab1* gene silencing on the expression and localization of Lc3b, Lamp2a, mTOR, Grp78, and Hsc70.

## 2. Materials and Methods

### 2.1. Ethics

The Shiga University of Medical Science Guidelines for the Care and Use of Laboratory Animals permitted the use of animals. The University of Split School of Medicine’s Ethical Committee gave its approval to the study, which was carried out in accordance with Directive 2010/63/EU on the protection of animals used for scientific purposes (protocol code no. 2181-198-03-04-23-0073; 27 September 2023).

### 2.2. Generation of Dab1 Null Conventional Mutants and Sample Collection

*Yotari* (*yot*) and C57BL/6N (wt) mice, colonies genetically similar to each strain [42], were bred and maintained individually in groups of three to four in standard polycarbonate cages with free access to food and tap water in a temperature-controlled (23 ± 2 °C) environment. For every recorded time point, three mice were used for each genotype (*yotari* and wt). Twelve hours of artificial light and twelve hours of darkness made up the photoperiod. For genotyping, the following PCR primers listed were utilized: *yotari*: GCCCTTCAG-CATCACCATGCT and CAGTGAGTACATATTGTGTGAGTTCC; wild-type *Dab1* locus: GCCCTTCAGCATCACCATGCT and CCTTGTTTCTTTGCTTTAAGGCTGT [43]. Pregnant mice were sacrificed at 13.5 and 15.5 days of pregnancy and embryos/fetuses were fixed in 4% buffered paraformaldehyde.

### 2.3. Immunofluorescence

After being fixed, the tissue was embedded in paraffin blocks, cut into 5 µm thick slices serially, and then graded ethanol solutions (Sigma-Aldrich, St. Louis, MO, USA) were used to mount the slices on glass slides. To ensure adequate tissue preservation, hematoxylin and eosin staining was applied to every tenth segment. After being deparaffinized in xylol, the mounted tissue sections were rehydrated in distilled water and graded ethanol. They were then heated in a water steamer for 20 min at 95 °C in a sodium citrate buffer (pH 6.0) and an unspecific secondary antibody reaction was blocked using blocking buffer (ab64226, Abcam, Cambridge, UK) for 30 min. In a humidity chamber, the samples were incubated with primary antibodies (Table 1) over night. After rinsing them with PBS the next day, they were incubated for an hour with the appropriate secondary antibodies (Table 1). Following a final PBS wash, the nuclei were stained with 4,6-diamidino-2-phenylindole (DAPI) and the samples were cover-slipped using Immu-Mount (Thermo Shandon, Pittsburgh, PA, USA). No staining was observed when primary antibodies were omitted in the immunofluorescence protocol.

Before the pre-adsorption test, a blocking solution was used to properly dilute each primary antibody. The combination was applied to the sections after the appropriate peptide antigen was introduced. The results showed no antibody staining. The absence of primary antibodies in the immunofluorescence technique did not result in false-positive results or non-specific secondary antibody binding.

### 2.4. Immunofluorescence Signal Quantification

To measure the immunofluorescence of the proteins under analysis, we computed the area percentage of the fluorescence signal in the obtained images. Each image was processed using the following steps. First, the background signal was removed using the “levels” tool in Adobe Photoshop, version 21.0.2 (Adobe, San Jose, CA, USA). After the epithelium was selected with the lasso tool and isolated from the lamina propria, it was then clipped from the original image and placed onto a blank image with the same dimensions. Next, the red color channel was eliminated in order to isolate the green signal in the two distinct images using ImageJ software version 1.530 (NIH, Bethesda, MD, USA). After making duplicates of the images, one of them was given the median filter. To isolate the positive signal, the filtered images were subtracted from the unfiltered images. The “triangle” option was used to threshold the final images, after which they were transformed to 8-bit images. The “analyze particles” function was used to determine the area percentage of the thresholded images. The measured area percentage was less than the actual area percentage because there were areas in all of the evaluated photos that had no tissue. To change the area percentage value, we counted the total pixels (px) in each image and the amount of empty pixels using Adobe Photoshop’s magic wand tool. The adjusted area percentage was calculated using the following formula, and it was then used in the statistical analysis:

$$Corrected\;area\;percentage=\frac{Uncorrected\;area\;percentage\;\times \;total\;px}{total\;px-empty\;space\;px,}$$

In addition, four levels of semi-quantitative evaluation were used to assess the staining intensity of different lung structures: no reactivity (−), mild reactivity (+), moderate reactivity (++), and strong reactivity (+++). The images were assessed independently by three researchers who were blinded to the time points and the mouse strain. When the interpreter agreement was tested using interclass correlation analysis, the results revealed high agreement with a coefficient of >0.75 [44].

### 2.5. Statistical Analysis

The software GraphPad Prism version 9.0.0 (GraphPad Software, San Diego, CA, USA) was used to conduct the statistical analysis. The mean and standard deviation of the calculated percentages are displayed for each result. The Shapiro–Wilk test was used to check whether the data distribution was normal. Tukey’s post hoc test combined with two-way analysis of variance (ANOVA) was utilized to determine the statistical significance of the differences in protein expression across the examined sample groups. At *p* < 0.05, statistical significance was established.

## 3. Results

In the lungs of wt and *yotari* mice, the immunoexpression of Lc3b, Grp78, Hsc70, mTOR, and Lamp2a was observed in the embryonic respiratory epithelium and mesenchyme. Each genotype included at least four specimens at both timepoints, E13.5 and E15.5.

### 3.1. Lc3b Expression

**Figure 1:**
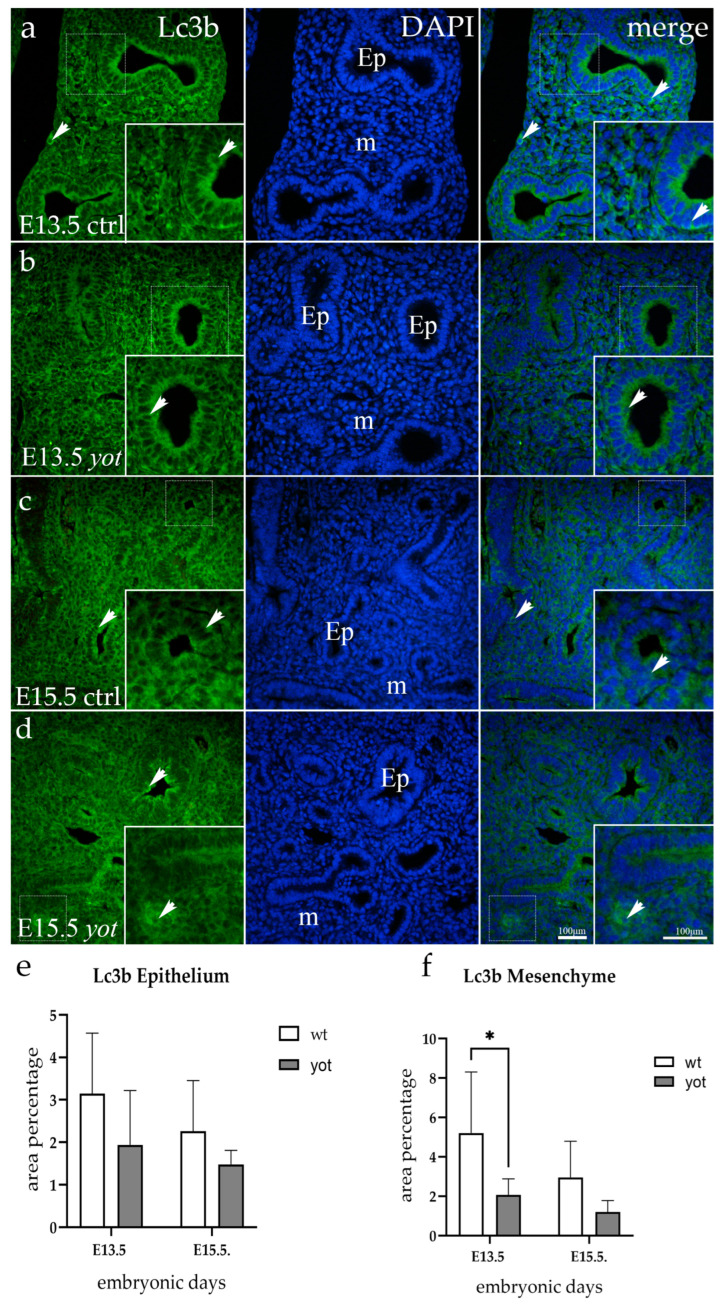
Immunofluorescent staining was performed on developing wild-type (ctrl) (**a**,**c**) and *yotari* (*yot*) (**b**,**d**) mouse lungs with Lc3b marker alongside 4′,6-diamidino-2-phenylindole (DAPI) nuclear staining. The expression pattern of Lc3b in the respiratory epithelium (Ep) and mesenchyme (m) was highlighted with arrows. The inserts depict the most prominent protein expression areas, corresponding to the dashed boxes. Images were captured at a total magnification of 400×, with a scale bar of 100 μm applicable to all images. The distribution of Lc3b-positive cells in the respiratory epithelium (Ep) and mesenchyme (m) of ctrl and *yot* lungs at embryonic days E13.5 and E15.5 is illustrated in graphs (**e**,**f**), respectively. Data are represented as mean ± SD and were analyzed using a two-way ANOVA test followed by Tukey’s multiple-comparison test (* *p* < 0.05 denotes significant differences). Ten substructures were evaluated at each time point.

The respiratory epithelium in E13.5 wt mice had a moderate diffuse signal, primarily situated apically in the cytoplasm, while the immunoexpression in the mesenchyme was sporadic and punctate in a small percentage of interstitial cells (Figure 1a). Upon examination of *yotari* mice, pulmonary epithelial cells displayed an identical signaling pattern as wt mice, while the mesenchyme showed no reactivity (Figure 1b). The wt mice showed stronger immunoexpression in the mesenchyme when these two genotypes were compared at this particular timepoint (*p* = 0.0128).

For E15.5 animals, the staining pattern remained the same, although it was less intense, particularly in epithelial cells. The immunoexpression was mostly apically localized in both genotypes and there was no basolateral signal (Figure 1c,d).

Semi-quantitative analysis for E13.5 exhibited that wt mice showed mild intensity in the epithelium and moderate intensity of the signal in the mesenchyme, whereas *yotari* showed either no reactivity or mild reactivity in the evaluated tissues. At E15.5, a mild signal was observed only in the mesenchyme of wild-type mice (Table 2).

### 3.2. Grp78 Expression

**Figure 2:**
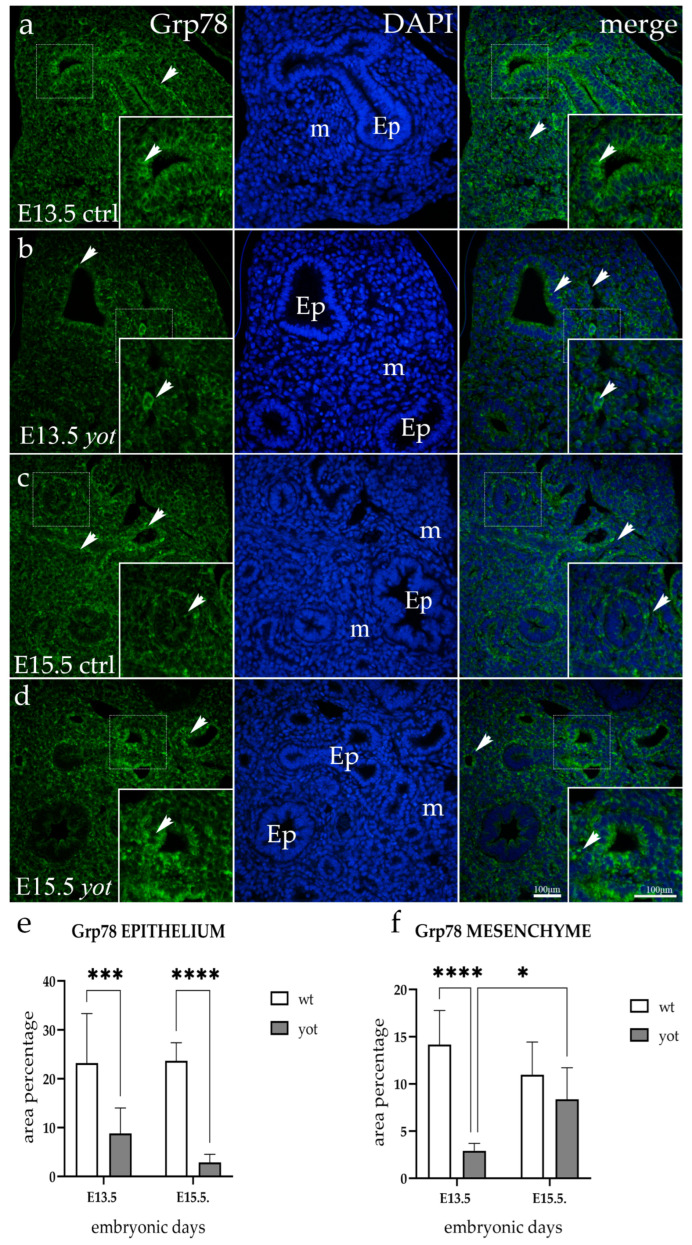
Immunofluorescent staining was performed on developing wild-type (ctrl) (**a**,**c**) and *yotari* (*yot*) (**b**,**d**) mouse lungs with Grp78 marker alongside 4′,6-diamidino-2-phenylindole (DAPI) nuclear staining. The expression pattern of Grp78 in the respiratory epithelium (Ep) and mesenchyme (m) is highlighted with arrows. The inserts depict the most prominent protein expression areas, corresponding to the dashed boxes. Images were captured at a total magnification of 400×, with a scale bar of 100 μm applicable to all images. The distribution of Grp78-positive cells in the respiratory epithelium (Ep) and mesenchyme (m) of ctrl and *yot* lungs at embryonic days E13.5 and E15.5 is illustrated in graphs (**e**,**f**), respectively. Data are represented as mean ± SD and were analyzed using a two-way ANOVA test followed by Tukey’s multiple-comparison test (* *p* < 0.05, *** *p* < 0.0001, **** *p* < 0.00001, denotes significant differences). Ten substructures were evaluated at each time point.

Grp78 staining showed a strong punctate signal in the cytoplasm of the respiratory epithelium of E13.5 wild-type animals, encircling the whole nucleus. The immunoexpression in the mesenchyme was diffuse and mainly located next to the epithelial cells (Figure 2a). *Yotari* mice showed weaker reactivity in the mesenchyme (*p* < 0.0001) and epithelium (*p* = 0.0006) compared to wt mice and the signal was mostly centered apically in the cytoplasm (Figure 2b).

Both genotypes displayed a strong punctate mesenchymal signal at E15.5 (Figure 2c,d), but the wt mouse epithelial staining was significantly stronger and similar to that of the E13.5 timepoint (*p* < 0.0001).

Control (wt) E13.5 specimens showed moderate staining for both examined structures in the semi-quantitative analysis, whereas same-age *yotari* specimens showed a mild signal. While both genotypes at E15.5 showed a mild mesenchymal signal, the mice in the control group displayed a moderate one in epithelial cells (Table 2).

### 3.3. Hsc70 Expression

**Figure 3:**
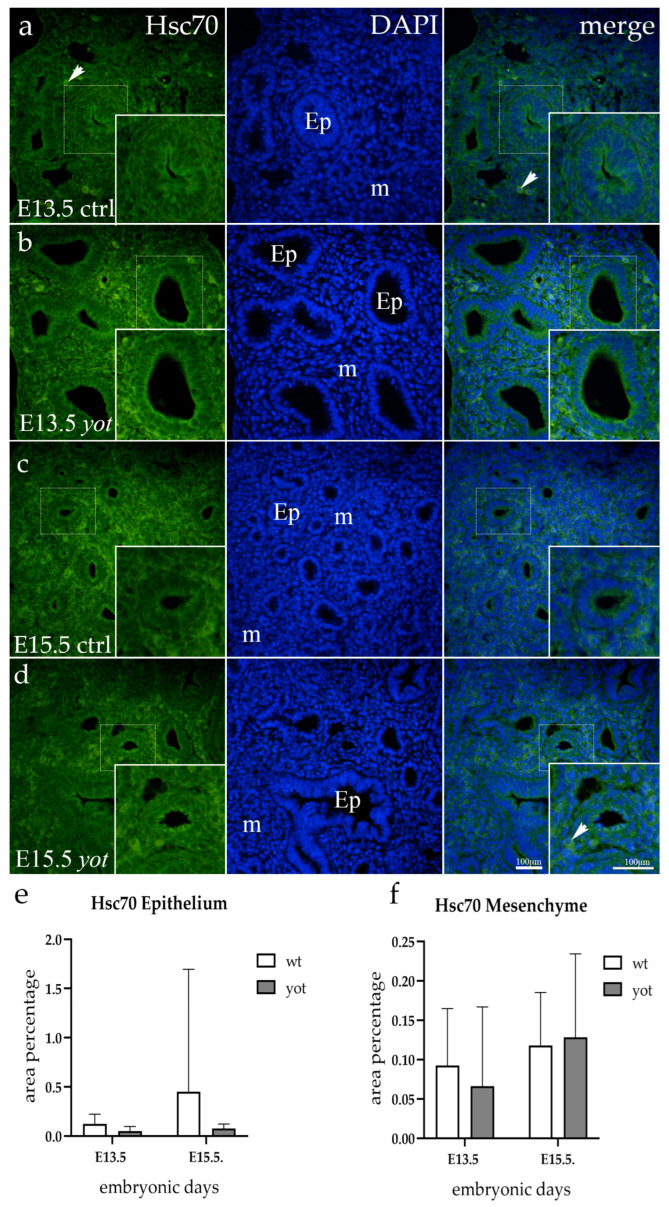
Immunofluorescent staining was performed on developing wild-type (ctrl) (**a**,**c**) and *yotari* (*yot*) (**b**,**d**) mouse lungs with Hsc70 marker alongside 4′,6-diamidino-2-phenylindole (DAPI) nuclear staining. The expression pattern of Hsc70 in the respiratory epithelium (Ep) and mesenchyme (m) was highlighted with arrows. The inserts depict the most prominent protein expression areas, corresponding to the dashed boxes. Images were captured at a total magnification of 400×, with a scale bar of 100 μm applicable to all images. The distribution of Hsc70-positive cells in the respiratory epithelium (Ep) and mesenchyme (m) of ctrl and *yot* lungs at embryonic days E13.5 and E15.5 is illustrated in graphs (**e**,**f**), respectively. Data are represented as mean ± SD and were analyzed using a two-way ANOVA test followed by Tukey’s multiple-comparison test.Ten substructures were evaluated at each time point.

In the epithelium of both wt and *yotari* mice at the E13.5 and E15.5 developmental phases, Hsc70 staining was completely absent. On the other hand, mesenchyme from both genotypes showed a mild punctate expression of Hsc70 at E13.5, with the signal increasing at the E15.5 timepoint (Figure 3).

Semi-quantitative analysis suggests mild reactivity was present in mesenchyme for every observed group of animals (Table 2).

### 3.4. Lamp2a Expression

**Figure 4:**
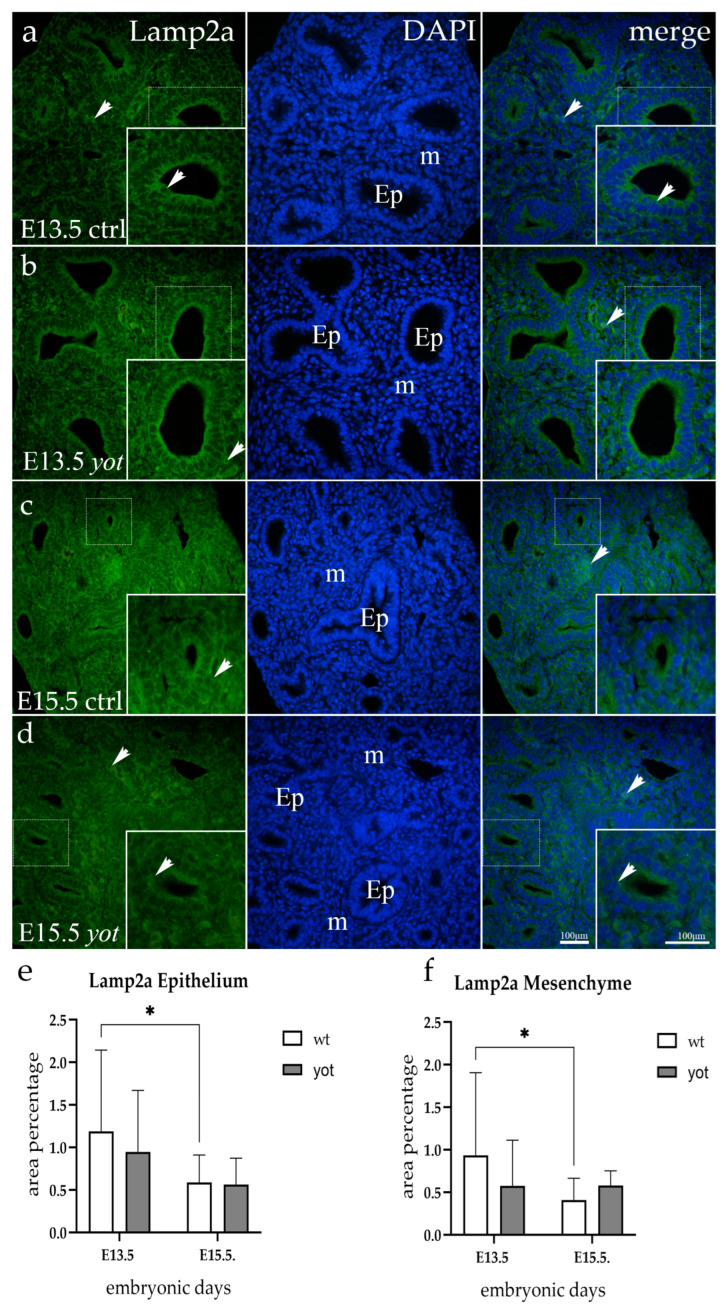
Immunofluorescent staining was performed on developing wild-type (ctrl) (**a**,**c**) and *yotari* (*yot*) (**b**,**d**) mouse lungs with Lamp2a marker alongside 4′,6-diamidino-2-phenylindole (DAPI) nuclear staining. The expression pattern of Lamp2a in the respiratory epithelium (Ep) and mesenchyme (m) was highlighted with arrows. The inserts depict the most prominent protein expression areas, corresponding to the dashed boxes. Images were captured at a total magnification of 400×, with a scale bar of 100 μm applicable to all images. The distribution of Lamp2a-positive cells in the respiratory epithelium (Ep) and mesenchyme (m) of ctrl and *yot* lungs at embryonic days E13.5 and E15.5 is illustrated in graphs (**e**,**f**), respectively. Data are represented as mean ± SD and were analyzed using a two-way ANOVA test followed by Tukey’s multiple comparison test (* *p* < 0.05 denotes significant differences). Ten substructures were evaluated at each time point. As for the semi-quantitative evaluation, a mild signal was observed in the epithelium of both genotypes in younger developmental-stage animals. Somewhat weaker mesenchyme reactivity was described for all groups except for E13.5 *yotari* mice, where it was absent (Table 2).

The wt and *yotari* specimens’ epithelial cells displayed fluorescence of the Lamp2a at E13.5 embryonic stage. The control group’s signal was strong and was mostly concentrated in the apical cytoplasm. This basolateral expression was nearly nonexistent in *yotari* mice (Figure 4a,b). At E15.5, the same epithelial immunoexpression pattern was present, though significantly less intensely (Figure 4c,d). All observed animals showed weak, punctate signals in the mesenchyme, with no significant difference between the two genotypes.

### 3.5. mTOR Expression

**Figure 5:**
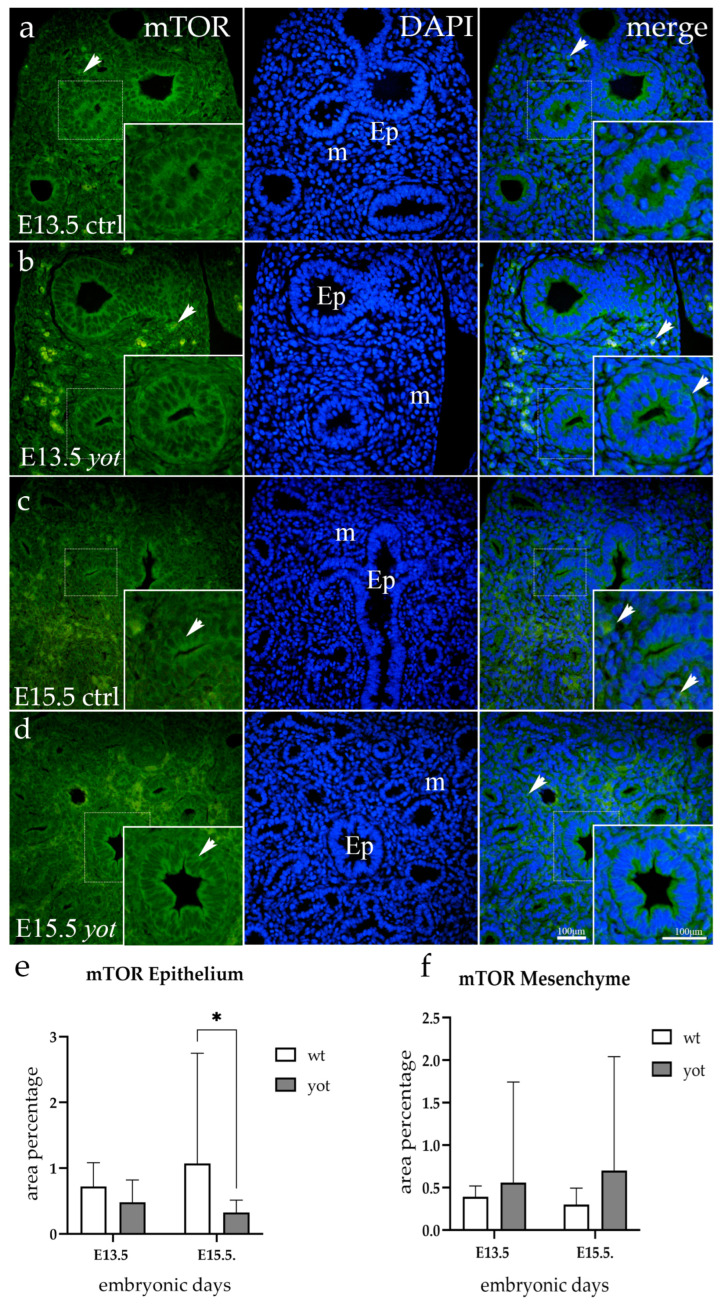
Immunofluorescent staining was performed on developing wild-type (ctrl) (**a**,**c**) and *yotari* (*yot*) (**b**,**d**) mouse lungs with mTOR marker alongside 4′,6-diamidino-2-phenylindole (DAPI) nuclear staining. The expression pattern of mTOR in the respiratory epithelium (Ep) and mesenchyme (m) is highlighted with arrows. The inserts depict the most prominent protein expression areas, corresponding to the dashed boxes. Images were captured at a total magnification of 400×, with a scale bar of 100 μm applicable to all images. The distribution of mTOR-positive cells in the respiratory epithelium (Ep) and mesenchyme (m) of ctrl and *yot* lungs at embryonic days E13.5 and E15.5 is illustrated in graphs (**e**,**f**), respectively. Data are represented as mean ± SD and were analyzed using a two-way ANOVA test followed by Tukey’s multiple-comparison test (* *p* < 0.05 denotes significant differences). Ten substructures were evaluated at each time point. Semi-quantitative analysis revealed a mild epithelial signal for wt and a mild mesenchymal signal for *yotari* mice at E13.5. At E15.5, wt mice exhibited moderate reactivity in the epithelium, while *yotari* mice showed mild mesenchymal reactivity (Table 2).

In the epithelium of the wt mice, there was a noticeable strong perinuclear staining both apically and basolaterally, which increased in later developmental stages. *Yotari* mice displayed the same signaling pattern; however, there was a noticeable variation in intensity between the two genotypes at E15.5 (*p* = 0.041). Mesenchymal staining was more intense in mutant specimens, but the difference was not statistically significant (Figure 5).

## 4. Discussion

Since lung diseases cause major mortality and morbidity worldwide, understanding the complex process of lung development has the potential for significant human impact and holds the promise that investigators can use this knowledge to aid lung repair and regeneration [45]. Congenital lung malformations, such as congenital pulmonary adenomatoid malformations, bronchopulmonary sequestrations, congenital lobar emphysema, and bronchogenic cysts, are one of the most common congenital disabilities in children, while other chronic lung respiratory diseases, such as chronic obstructive pulmonary disease (COPD) or asthma, are the third leading cause of death worldwide [46]. Since autophagy plays an important role in lung homeostasis through several mechanisms, including the preservation of mitochondrial homeostasis and the removal of misfolded proteins [47], in this study, we hypothesized that *Dab1* knockout animals would exhibit a distinct pattern of autophagy markers when compared to wt animals. In order to confirm our theory, we investigated different expression patterns of Lc3b, Grp78, Hsc70, Lamp2a, and mTOR in the lungs during the embryonic developmental period using a *yotari* mouse model.

When compared to *yotari*, wt mice in our study displayed greater Lc3b immunoexpression. The signal was diffuse and mostly located in the cytoplasm in the epithelium, while it was sporadic in the mesenchyme. These findings partially align with previous research indicating that mice with a reduced expression of Lc3b exhibit greater resilience to modifications in autophagic functions, which might be a reflection of poor lung development in these animals. Additionally, this work showed that epithelial and mesenchymal cells exhibit strong Lc3b immunoexpression in the early stages of embryonic development. An increased susceptibility to pulmonary injury and fibrosis was evident in older animals. In a prior study, autophagosome formation, Lc3b production, and its activation were induced using mouse lung vascular tissue from an animal model of hypoxia-induced pulmonary hypertension. These preliminary findings reveal Lc3b activation and link autophagy to the pulmonary vasculature’s general response to hypoxic stress. Thus, autophagic proteins have been implicated in both protective and maladaptive roles, depending on the stress model [48,49].

In contrast to previous research that showed higher Grp78 expression in the epithelial cells of later stages of mouse lung development, our study demonstrated significant Grp78 expression in both epithelial and mesenchymal lung cells during the early stages of embryonic development in wt mice [38]. As compared to mesenchyme, where there was enhanced immunoexpression surrounding epithelial cells, a punctate epithelial signal was primarily localized in the cytoplasm. Similar studies show the important role of these expressions in lung diseases such as pulmonary fibrosis and COPD [50]. Alveolar macrophage accumulation is strongly correlated with the severity of the disease in COPD patients, and chronic lung inflammation plays a crucial role in the pathophysiology of the disease [51]. The ER is the intracellular site of maturation and folding of secretory and membrane proteins. Disturbances in the ER luminal environment, genetic mutations, viral infection, and other insults cause misfolded proteins to accumulate [52]. Our findings are, however, in complete agreement with the research conducted by Lam et al., which demonstrated that chronic lung inflammation and emphysema are caused by insufficient Grp78-mediated autophagy. These findings indicate that Grp78 is critical for the development of early embryos and that its absence may result in a number of pro-apoptotic pathways and early embryonic lethality [51,53].

While mesenchyme displayed a modest punctate expression of Hsc70 at E13.5, with the signal increasing at the E15.5 timepoint, epithelium showed a complete absence of Hsc70 staining. A study by Deutschman and Weiss showed that lung-specific delivery of Hsp70 prevents acute respiratory distress syndrome. In this research, aged septic mice with genetic mutations had increased epithelial apoptosis and increased systemic inflammation compared to aged septic wt mice [54]. These findings might point to CMA’s potential preventive function against lung diseases. However, there was no apparent difference in Hsc70 immunoexpression between the two genotypes that were included in our study. According to our results, the expression of Lamp2a is more pronounced in earlier control animals, suggesting potential association of this autophagic marker with aging. According to the report of recent studies, CMA pathways are significantly impacted by aging, primarily via the nuclear factor erythroid 2-related factor 2 (Nrf2) positive-feedback regulation network, where Nrf2 regulates cellular resistance to oxidants. Oxidative stress triggers CMA, which in turn increases Nrf2’s transcriptional activity. Enhanced Nrf2 stimulates the expression of the Lamp2a gene, which increases CMA activity, closing the positive-feedback loop [55,56]. The first direct evidence of CMA decline during ageing was presented by Cuervo et al. They found a progressive decrease in Lamp2a and CMA activity levels in lysosomes. They also demonstrated that the reduced Lamp2a was due to the altered dynamics and stability of Lamp2a in the lysosomal compartment, resulting in its increased mobilization to microdomains and subsequent degradation in the lysosome [57]. These findings suggest that lung epithelial cells’ apoptosis due to reduced CMA regulated by Nrf2 may be directly associated with the development of COPD. Consequently, it is possible that using CMA as an antiapoptotic therapy approach for COPD could prove to be beneficial [58].

Extensive studies have established a dominant role for mTOR in regulating cellular growth and metabolism in response to growth factors and nutrients and reveal that the mTOR signaling pathway is implicated in the progression of cancer as well as the ageing process. Accumulating evidence suggests that mTOR and autophagy play critical roles in pulmonary diseases in mouse airway epithelial cells [59]. In our study, the expression of mTOR in epithelial cells during initial and late embryonic development was more pronounced in wt mice. Although mTOR expression in mesenchymal cells is more prominent in *yotari* animals, these findings show that mTOR is essential for cell development and proliferation in both early mouse embryonic cells. Mice with a genic mutation that causes alveolar epithelial cells or lung vascular cells to become overactive in mTOR exhibit cellular senescence, mimicking the lung changes associated with COPD [60]. It is demonstrated that aberrant activation of the Akt-mTOR pathway in the lung epithelium contributes to the pathophysiology of respiratory distress syndrome [61]. Because mTOR is an essential negative regulator of autophagy [62], its activation may decrease autophagy, leading to cell senescence, but also suggesting an important protective role of autophagy. This theory is supported by the reduction in autophagy proteins in the lungs of TSC1-KO mice [63]. In contrast to other pro-autophagic markers that showed an increased signal in wt animals, mTOR expression in mesenchyme was stronger in mutant specimens in our study. This suggests that this marker may have a protective role in lung development, as it was previously confirmed for other organs [64].

The important role of Lc3b, Grp78, Hsc70, Lamp2a, and mTOR markers in comprehending lung processes can be seen by their dynamic and expression patterns in both *yotari* and wt mice. It is possible to speculate that the buildup of unfolded or misfolded proteins brought on by *Dab1* gene silencing increases the expression of chaperones and other autophagic biomarkers, but also increases the expression of negative autophagy regulators, such as mTOR, a master regulator of cellular metabolism.

In conclusion, our study highlights the importance of autophagy in lung development and suggests that it has a protective role in preventing programmed cell death. Since each of the identified proteins is important for autophagy, they are suitable biomarkers to quantify activity of this process. Further exploration of these proteins could potentially lead to the development of targeted therapies for lung diseases characterized by dysregulated autophagy. Moreover, understanding the intricate mechanisms underlying autophagy regulation may offer insights into novel strategies for enhancing lung health and combating respiratory conditions.

## Figures and Tables

**Table 1:** Antibodies used for immunofluorescence.

Antibodies		Host	Dilution	Source
Primary	Anti-Lc3b/ab48394	Rabbit	1:100	Abcam (Cambridge, UK)
	Anti-Grp78/PA5-19503	Rabbit	1:300	Thermo Fisher Scientific (Waltham, MA, USA)
	Ani-Hsc70/ab51052	Rabbit	1:50	Abcam (Cambridge, UK)
	Anti-Lamp2a/ab18528	Rabbit	1:100	Abcam (Cambridge, UK)
	Anti-mTOR/ab32028	Rabbit	1:100	Abcam (Cambridge, UK)
Secondary	Anti-Rabbit IgG, Alexa Fluor^®^ 488, 711-545-152	Donkey	1:300	Jackson Immuno Research Laboratories, Inc. (Baltimore, PA, USA)

**Table 2:** Staining intensity of certain antibodies at embryonic days E13.5 and E15.5 in the lungs of yotari and wild-type mice.

Embryonic Day (E)	Animal	Structure	Antibody				
			Lc3b	Grp78	Hsc70	mTOR	Lamp2a
E13.5	wild-type	Ep	+	++	−	+	+
		m	++	++	+	−/+	−/+
	*yotari*	Ep	−/+	−/+	−	−/+	+
		m	−/+	−/+	+	+	−
E15.5	wild-type	Ep	−/+	++	−/+	++	−/+
		m	+	+	+	−/+	−/+
	*yotari*	Ep	−/+	−/+	−	−/+	−/+
		m	−/+	+	+	+	−/+

++ moderate reactivity; + mild reactivity; − no reactivity; Ep—respiratory epithelium; m—mesenchyme; E—day of embryonic development; 1B-light chain 3 (Lc3b), glucose-regulated protein 78 (Grp78), heat shock cognate 71 kDa protein (Hsc70), mammalian target of rapamycin (mTOR) and lysosomal-associated membrane protein 2A (Lamp2a).

## Data Availability

All data and materials are available upon request.
